# Analytical Techniques for the Determination of Paracetamol and Ibuprofen Combination

**DOI:** 10.1155/jamc/6822390

**Published:** 2025-07-30

**Authors:** Imad O. Abu Reid, Sayda M. Osman, Somia M. Bakheet

**Affiliations:** Department of Pharmaceutical Chemistry, Faculty of Pharmacy, International University of Africa, Khartoum, Sudan

**Keywords:** determination, dosage form, ibuprofen, paracetamol

## Abstract

The fixed-dose combination (FDC) of ibuprofen (IBU) and paracetamol (PAR) has emerged as a preferred option in pain management, owing to its distinct practical advantages. Both drugs have well-documented efficacy and safety profiles, providing synergistic pain relief through complementary mechanisms of action. IBU not only offers central analgesic effects but also inhibits cyclooxygenase (COX) enzymes, particularly COX-1 and COX-2, thereby reducing prostaglandin synthesis at the site of pain to deliver both analgesic and anti-inflammatory benefits. Despite the growing use of this combination, a comprehensive review focusing on the analytical methods for its determination has not yet been published. This review aims to bridge that gap by presenting an extensive compilation of documented analytical methods for the quantification of IBU and PAR in both bulk and pharmaceutical formulations. It serves as a valuable resource for researchers and professionals seeking detailed insights into the diverse techniques employed for accurate and precise analysis of these FDCs. Through a systematic search of major scientific databases, including Science Direct, Springer Link, PubMed, Scopus, and Google Scholar, the review identifies the most commonly utilized methods, such as high-performance liquid chromatography (HPLC), gas chromatography (GC), high-performance thin-layer chromatography (HPTLC), ultraviolet (UV)/Visible spectrophotometry, Fourier-transform infrared spectroscopy (FTIR), and micellar electrokinetic chromatography (MEKC). Notably, HPLC and UV/Visible spectrophotometry were the most frequently reported, each accounting for 37.9% of studies. By consolidating these analytical approaches, this review highlights the state-of-the-art methodologies available for the determination of IBU/PAR FDCs and underscores its novel contribution as a definitive reference for future research and development in this field.

## 1. Introduction

Acute pain results from tissue damage, whether from accidental injury or surgery. Postoperative pain, in particular, is caused by inflammation from surgical tissue injury. Effectively managing postoperative pain and inflammation is vital for patient care and contributes to the cost-effective use of healthcare resources. Proper pain management after surgery ensures patient satisfaction and helps reduce the time spent in the hospital or at home unable to perform normal activities.

For postoperative pain relief, analgesics include “mild” or Step 1 options such as paracetamol (PAR) (acetaminophen) and nonsteroidal anti-inflammatory drugs (NSAIDs) such as ibuprofen (IBU) and celecoxib. “Moderate” or Step 2 analgesics include weaker opioids such as codeine, while “strong” or Step 3 analgesics involve potent opioids such as oxycodone and fentanyl [1].

Opioids have traditionally been used to manage pain during and immediately after surgery due to their parenteral administration and adjustable dosages for immediate pain relief. Oral opioids, while less commonly used alone, are often combined in fixed doses with other medications such as PAR or IBU [2]. Despite their effectiveness, the global rise in opioid misuse has become an increasingly serious issue [3].

To address the opioid overuse epidemic and reduce opioid-related deaths, it is essential to explore alternative pain management strategies, such as nonprescription analgesics. The fixed-dose combination (FDC) of IBU and PAR is gaining popularity for pain management due to several practical benefits. Both components of the IBU/PAR FDC have well-established efficacy and safety profiles, providing pain relief through complementary mechanisms. IBU not only offers central analgesic effects but also inhibits cyclooxygenase (COX) enzymes COX-1 and COX-2, reducing prostaglandin synthesis at the pain site and resulting in both analgesic and anti-inflammatory effects [4, 5].

In addition, the pharmacokinetics and pharmacodynamics of an FDC are similar to those of the individual drugs, but the combination may be more effective by leveraging the dual mechanisms of both ingredients, helping patients manage pain more effectively [6]. FDC formulations provide effective and tolerable pain relief with a consistent dosing schedule, often requiring lower doses of each component compared with taking the drugs separately [7, 8]. Patients might initially use one component of an FDC and add the other if necessary, but starting with the FDC can avoid this trial-and-error approach. Taking an FDC in a single pill is more convenient than taking multiple medications separately. Finally, FDC medications are more accessible and often available globally without a prescription [9].

Numerous analytical techniques have been employed for the determination of IBU alone in pharmaceutical formulations. These include chromatographic methods [10], ultraviolet (UV) spectrophotometry [11–13], colorimetric analysis [14, 15], electrochemical approaches [16, 17], nuclear magnetic resonance (NMR) spectroscopy [18, 19], and capillary zone electrophoresis (CZE) [20]. Similarly, various analytical approaches have been reported for the determination of PAR alone in pharmaceutical dosage forms, including spectrophotometric methods [21–27], electrochemical methods [28–37], and capillary electrophoresis (CE) [38, 39].

Analytical methods developed for FDCs provide several key advantages throughout pharmaceutical development, manufacturing, and quality control compared with methods designed for the individual analysis of each active ingredient separately. One of the primary benefits of these methods is their ability to enable simultaneous quantification of multiple APIs within a single analytical run. This ensures accuracy and efficiency while significantly reducing the consumption of reagents, solvents, and analytical time compared with separate analyses for each component [40]. This simultaneous approach directly supports cost-effectiveness, especially in high-throughput laboratories or settings with limited resources, aligning with the WHO guidance on affordable medicines quality control [41].

Moreover, analytical methods tailored for FDCs support improved quality assurance by confirming content uniformity and verifying the correct ratio of APIs in the final product. This is critical in minimizing dosage errors and batch variability [42]. Ensuring accurate dosage of each component contributes directly to enhanced patient safety and therapeutic efficacy, particularly in therapeutic areas such as HIV, TB, and malaria, where polytherapy is the standard [43].

From a regulatory compliance perspective, developing and validating robust analytical methods for FDCs is crucial. Regulatory bodies, including the ICH and WHO, require that methods used for pharmaceutical analysis meet stringent validation parameters such as specificity, accuracy, linearity, and precision [44]. Proper validation facilitates approval processes and ensures consistency across international markets.

Stability-indicating methods are especially important for FDCs due to the potential for inter-API interactions. These methods can detect degradation products of each API even in complex matrices, thus ensuring stability and product integrity throughout the shelf life [45]. This is consistent with the WHO guidance that recommends stability studies to ensure the long-term safety and efficacy of FDCs [46].

In terms of methodological flexibility, a variety of techniques can be used for FDC analysis, including high-performance liquid chromatography (HPLC), UPLC, CE, micellar electrokinetic chromatography (MEKC), and UV/Visible spectrophotometry. These techniques can be selected based on the properties of the APIs and the matrix, allowing for tailored solutions in both development and routine quality control settings [47].

Validated methods also enhance data integrity and traceability when implemented in laboratory information management systems (LIMS), fulfilling GLP and cGMP requirements [48]. This ensures that the data generated are reliable, audit-ready, and compliant with global data integrity expectations.

Finally, these analytical methods are vital in supporting bioequivalence and clinical studies, providing accurate measurements of API levels in plasma and dosage forms, which are essential for regulatory approval and therapeutic equivalence studies [49].

In summary, analytical methods for FDCs provide a comprehensive range of advantages: improved operational efficiency, cost-effectiveness, quality and safety assurance, regulatory compliance, and support for clinical development. These advantages make them indispensable tools in the successful formulation and lifecycle management of combination therapies aimed at addressing complex and chronic diseases globally.

To our knowledge, no comprehensive review on this topic has been published yet. The primary objective of this review is to compile the documented analytical methods for the determination of PAR combination IBU; in both bulk and pharmaceutical formulations. This review serves as a convenient reference for the diverse analytical techniques utilized to accurately and precisely quantify these drugs combinations.

This review consolidates references from 2007 onward, sourced through an extensive search of prominent scientific databases such as Science Direct, Springer Link, PubMed, Scopus, and Google Scholar. The focus was on identifying recent analytical methodologies for studying PAR and IBU combinations across various pharmaceutical formulations. The data is systematically categorized based on the analytical techniques employed, providing a clear and organized overview of the approaches used to analyze these binary mixtures. By presenting comprehensive insights from a wide range of published studies, the review serves as a valuable resource for pharmaceutical researchers and professionals, enabling them to easily access information on analytical methods relevant to their work.

## 2. Analytical Techniques

### 2.1. UV/Visible Spectrophotometric Methods

UV/Visible spectrophotometry is an analytical technique that measures the absorption of light by a substance in relation to its concentration. This method is based on the excitation of electrons within a molecule from a lower energy state to a higher energy state when exposed to light in the wavelength range of 200–800 nm. The direct relationship between the concentration of the analyte and the amount of absorbed light is mathematically described by the Beer-Lambert law as follows:

(1) $$\begin{array}{c} A=abc, \end{array}$$

where *A* is the absorbance (dimensionless), *b* is the path length of the light through the sample, measured in centimeters (cm), *c* is the concentration of the analyte, expressed in moles per liter (M) or grams per 100 mL (g/100 mL), and *a* is the molar absorptivity or absorption coefficient, with units dependent on the chosen concentration scale.

This fundamental law provides a simple yet powerful means of quantifying substances based on their light-absorbing properties, making UV/Visible spectrophotometry a versatile tool in various fields of chemical analysis.

Analyzing and controlling multicomponent formulations through direct spectrophotometry presents significant challenges for analytical chemists, primarily due to overlapping spectral bands. To overcome these obstacles, various spectrophotometric techniques have been developed to separate and quantify compounds with overlapping spectra. The effectiveness of these methods largely hinges on the extent of spectral overlap and the number of components in the mixture. For mixtures with minimal overlap, straightforward mathematical manipulations of spectral data are often sufficient [50]. In contrast, when overlap is extensive, comprehensive spectral analysis is required to accurately identify and quantify all components [51]. Precision in these analyses can be further improved by carefully selecting wavelength ranges, which minimizes collinearity and reduces spectral interference [52].

The experimental conditions and technical details of the spectrophotometric methods reported for the determination of IBU and PAR are summarized in Table 1.

The simultaneous equation method [53–58] offers straightforward and cost-effective approach for determining PAR and IBU in combination, delivering adequate sensitivity and a broad linear range. Its simplicity and ease of implementation make it particularly suitable for routine analysis, especially in laboratories located in low-income regions. Among the reported methods, the one developed by Gondalia et al. [57] is notably the most sensitive, with limits of detection (LODs) of 0.36 μg/mL for IBU and 0.28 μg/mL for PAR.

Methods based on ratio spectra manipulation, such ratio difference (RD), constant center (CC), and mean centering of ratio spectra (MCR), have also been employed for the determination of PAR and IBU in pharmaceutical dosage forms [64, 65]. While these techniques are effective in resolving overlapping spectral bands, their practicality for routine analysis is limited compared with simpler approaches. Simpler methods such as Q-absorbance ratio [53], dual wavelength [59], area first derivative and ratio first derivative [60], and area under the curve [61] have also been reported for analyzing PAR and IBU combination. These methods offer reasonable sensitivity and a broad linear range, making them more suitable for various routine applications.

### 2.2. HPLC

HPLC is an invaluable tool for evaluating the purity and quality of pharmaceutical formulations, especially when gas-liquid chromatography (GLC) is unsuitable due to the components' low volatility or poor thermal stability. Advances in selective adsorbents and improvements in the sensitivity of flow-through detectors, including spectrophotometric, fluorometric, and electrochemical types, have significantly broadened HPLC's applications in pharmaceutical analysis.

Chromatographic separation occurs due to differences in the affinity of compounds for the stationary phase versus the mobile phase. Compounds with stronger interactions with the stationary phase spend more time bound to it, resulting in slower movement through the chromatography system and eventual separation from compounds that interact less strongly. The differences in affinity between compounds are primarily caused by variations in their chemical properties, such as polarity, size, shape, and functional groups. These differences dictate how strongly each compound interacts with the stationary phase relative to the mobile phase, leading to variations in retention times and ultimately separation. The sample compounds can interact with the stationary phase through various intermolecular forces such as Van der Waals forces and dipole–dipole interactions.

Reversed-phase HPLC (RP-HPLC) is particularly prevalent for the quantification of PAR and IBU combinations in pharmaceutical dosage forms. Most methods utilize isocratic elution with reversed-phase columns (C_8_ or C_18_), and mobile phases typically consist of buffered organic solvent mixtures with carefully adjusted pH levels. The experimental conditions and technical details of the HPLC methods reported for the determination of IBU and PAR are summarized in Table 2.

Among these methods, the approach developed by Al-Salman [75] stands out for its exceptional sensitivity, offering a lower LOD and a wider linear range than other techniques. While all methods meet validation standards, the methodologies proposed by Borahan et al. [72], Makwana et al. [76], and Ashok Kumar et al. [77] are especially noteworthy for their stability-indicating capabilities. These were validated through forced degradation studies, demonstrating their effectiveness in identifying trace levels of degradation products and related substances alongside the intact analytes.

However, it is worth noting that only the method reported by Ashok Kumar et al. [77] underwent optimization using central composite design (CCD) whereas El-Kommos et al.'s [71] method was optimized using the obsolete, low efficiency, one-factor-at-a-time (OFAT) approach, while the other methods were validated without a formal optimization process. This distinction highlights potential areas for refinement in existing analytical methodologies.

### 2.3. High-Performance Thin Layer Chromatography (HPTLC)

The HPTLC separation of PAR and IBU is commonly conducted on silica gel 60F254 precoated plates under various chromatographic conditions.

Bhushan et al. [73] employed a mobile phase of toluene and acetonitrile in a 60:50 (v/v) ratio, with analyte detection at 247 nm. This method exhibited excellent linearity within the concentration range of 10–50 μg/mL for both analytes, with LODs of 1.6 μg/mL for IBU and 0.88 μg/mL for PAR, underscoring its sensitivity and precision.

In addition, Rele and Sawant [78] achieved the separation of IBU and PAR using a mobile phase comprising ethyl acetate, acetone, butanol, and ammonia in a 30:40:30:10 (v/v) ratio, with detection performed at 254 nm. The method demonstrated a working range of 1.2–6.0 μg/mL for PAR and 1.3–6.5 μg/mL for IBU, while Shirke et al. [79] utilized a mobile phase consisting of *n*-hexane, ethyl acetate, and glacial acetic acid in a 90:25:10 (v/v) ratio. Detection was carried out at 265 nm, with a linear working range of 10–50 μg/mL for PAR and 16–60 μg/mL for IBU.

### 2.4. Gas Chromatography (GC)

GC operates on the same fundamental principles as HPLC, with the primary distinction lying in the nature of the mobile phase. In GC, an inert carrier gas—such as helium, nitrogen, or argon—serves as the mobile phase, facilitating the transport of analytes through the chromatographic column without interacting with them. This gaseous mobile phase enables efficient separation of volatile and semivolatile compounds based on their differential affinities for the stationary phase and their varying boiling points, making GC particularly suitable for the analysis of thermally stable substances.

Zambakjian and Alhaj Sakur [80] developed a gas chromatographic method for analyzing IBU and PAR in bulk materials and pharmaceutical formulations. The method utilized a TRB-5 column (30 m length, 0.25 mm internal diameter, and 0.25 μm film thickness) with nitrogen as the carrier gas at a flow rate of 1 mL/min. The oven temperature was initially set at 180°C for 0.5 min and then increased at a rate of 15°C/min to a final temperature of 250°C. Both the injector and detector temperatures were maintained at 300°C. Detection was performed using a flame ionization detector (FID), with methyl paraben serving as an internal standard. The calibration curves for IBU and PAR were linear within the concentration range of 0.25–1.75 μg/mL, demonstrating the method's precision and reliability.

### 2.5. MEKC

CE encompasses a diverse array of separation techniques, each employing distinct mechanisms for analyte separation. CZE differentiates analytes based on their electrophoretic mobilities as they migrate through a buffer-filled capillary under an electric field.

El-Kommos et al. [81] developed a method for determining IBU and PAR using a fused silica capillary with a total length of 65 cm and an internal diameter of 50 μm. The separation was carried out with a borate buffer (20 mM, pH 9) containing 15% (v/v) methanol and 100 mM sodium dodecyl sulfate (SDS) under an applied voltage of 15 kV. Detection was performed at 214 nm. Various factors influencing the electrophoretic mobility of the seven studied drugs were investigated and optimized.

The method demonstrated linearity in the ranges of 200–2200 μg/mL for IBU and 30–1050 μg/mL for PAR, with LODs of 8.20 × 10^−3^ μg/mL for IBU and 0.01 μg/mL for PAR.

### 2.6. Fourier-Transform Infrared Spectroscopy (FTIR)

Infrared (IR) spectroscopy is an analytical technique that records the absorption of radiation within the IR region of the electromagnetic spectrum. An IR spectrophotometer measures the percentage of light transmitted through a sample as a function of wavelength. Key functional groups within a molecule are identified based on their characteristic absorption bands, which are largely independent of the molecule's overall structure. These absorption bands occur within specific, well-defined frequency ranges, though their exact positions may shift due to factors such as electronic effects, resonance, and hydrogen bonding.

Molecules often contain multiple bonds, each capable of undergoing various IR-active vibrational modes. As a result, IR spectra frequently exhibit numerous overlapping absorption bands, contributing to their complexity and making spectral interpretation a detailed and intricate process.

Two FTIR spectroscopy methods have been documented for the determination of PAR and IBU in pharmaceutical dosage forms. Nugrahani and Khalida [82] utilized the N–H stretching region (3289–3220 cm^−1^) for PAR and the carbonyl group absorption range (1847–1758 cm^−1^) for IBU quantification. The method exhibited linearity across concentration ranges of 1%–6% w/w for PAR and 1.5%–4% w/w for IBU. The LOD were calculated to be 3.249 × 10^−5^% w/w for PAR and 6.652 × 10^−4^% w/w for IBU, demonstrating the method's high sensitivity and reliability, while Ali et al. [83] utilized the stretching range of 1781–1683 cm^−1^ for IBU and 1630–1530 cm^−1^ for PAR in their quantification method. The LOD for both PAR and IBU was determined to be 0.001 mg/g, highlighting the method's precision and sensitivity. The LOD values clearly indicate that the method developed by Nugrahani and Khalida [82] is significantly more sensitive than the one reported by Ali et al. [83].

Both methods are environmentally friendly as they eliminate the need for solvent use.

## 3. Conclusions

**Figure 1:**
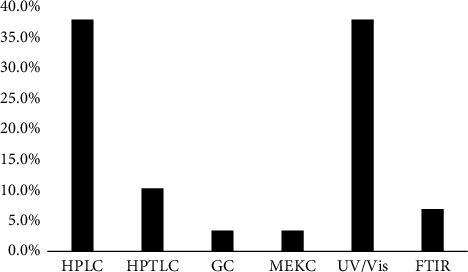
Graph displaying the % ratio of the analytical methods used for the simultaneous estimation of paracetamol and ibuprofen combinations.

This study provides a comprehensive review of the analytical methods commonly used for the determination of PAR and IBU combinations. It encompasses a wide range of techniques applied to these compounds in bulk materials and pharmaceutical dosage forms, with a particular focus on advancements reported from 2007 to the present. The most frequently utilized methods include HPLC, GC, HPTLC, UV/Visible spectrophotometry, FTIR, and MEKC. As illustrated in Figure 1, HPLC and UV/Visible spectrophotometry emerged as the most widely adopted techniques, each accounting for 37.9% of studies. These were followed by HPTLC (10.3%), FTIR (6.9%), and GC and MEKC, which contributed 3.4% each.

Future research could focus on optimizing analytical methods following the quality-by-design approach and developing more methods using ecofriendly solvents for determining PAR combinations with IBU in pharmaceutical preparations.

The pattern of analytical techniques employed in this review closely parallels that observed in our previously published review on the metformin–thiazolidinedione combination [84].

## Figures and Tables

**Table 1:** Spectrophotometric methods used for the determination of paracetamol and ibuprofen combination.

No.	Technique	Wavelengths nm	Solvent	LOD (μg/mL)	Linear range (μg/mL)	Reference
1	Simultaneous equation	256 and 222.4	Methanol	NA	5–30 both	[53]
	*Q*-ratio method	226.4 (isosbetic)				
		256 IBU				

2	Simultaneous equation	220 and 240	Ethanol	0.214 PAR	1–15 PAR	[54]
				0.600 IBU	2–20 IBU	

3	Simultaneous equation	222 and 243	Phosphate buffer (pH 7.2)	NA	3.2–4.8 IBU	[55]
					4–6 PAR	

4	Simultaneous equation	257 and 222	0.1 N NaOH	0.198 PAR	1–10 PAR	[56]
				0.800 IBU	1–12 IBU	

5	Simultaneous equation	224 and 248	Methanol	0.360 IBU	4–14 IBU	[57]
				0.280 PAR	2–12 PAR	

6	Simultaneous equation	220 and 240	0.1 N NaOH	0.610 PAR	10–60 PAR	[58]
				0.199 IBU	2–50 IBU	

7	Dual wavelength	220 and 231 PAR	Ethanol	NA	6-12 both	[59]
		241 and 255 IBU				

8	First derivative	230 IBU	Methanol	NA	5–100 IBU	[60]
		290 PAR				
	First derivative ratio	280 IBU				
		290 PAR			10–100 PAR	
	Classical least squares	200 to 30				
	Principal component regression					

9	Area under curve	244–254 PAR	Methanol	0.690 PAR	3–9 PAR	[61]
		220–230 IBU		0.680 IBU	5–13 IBU	

10	H-point standard addition	610	Alkaline KMnO_4_	0.160 PAR	1–20 PAR	[62]
				0.550 IBU	5–25 IBU	

11	Derivative transform	210–290	Phosphate buffer pH 7.2	NA	12–32 IBU	[63]
	Wavelet transform				20–40 PAR	

12	Ratio difference	210.6 and 216.4 IBU	Methanol	0.630 PAR	2–20 PAR	[64]
				0.985 IBU		
	Constant center	236.0 and 248.0 PAR		0.097 PAR		
				0.010 IBU	2–50 IBU	
	Mean centering	240 IBU		0.590 PAR		
		237 PAR		0.189 IBU		

13	Mean centering	244 PAR	pH 7.2 phosphate buffer-ethanol solvent mixture	NA	3–9 PAR	[65]
		221 IBU			5–13 IBU	

**Table 2:** High-performance liquid chromatographic methods used for the analysis of ibuprofen and paracetamol combination.

No.	Column	Mobile phase	Detection λ (nm)	Working range (μg/mL)	LOD (μg/mL)	Reference
1	C_18_, 25 cm × 4.6 mm, 5 µm	Methanol: 0.025 M phosphate buffer pH 3.0 (80:20 v/v). The flow rate was 1.0 mL/min	225	5.84–8.76 PAR	NA	[66]
	Caffeine internal standard			7.09–10.6 IBU		

2	C_18_, 25 cm × 4.6 mm, 5 µm	0.05 M sodium dihydrogen phosphate (65:35 v/v) as mobile phase, at a flow rate of 1.0 mL/min	230	50–400 PAR	0.40 PAR	[67]
				20–160 IBU	0.50 IBU	

3	C_8_, 25 cm × 4.6 mm, 5 µm	Acetonitrile:water:trimethylamine (pH 3.5 adjusted by using orthophosphoric acid) in the ratio of 60:40:0.1 (v/v/v), at a flow rate of 1.0 mL/min	220	6.5–32.5 PAR	0.23 PAR	[68]
				8–40 IBU	0.50 IBU	

4	C_8_, 25 cm × 4.6 mm, 5 µm	0.045 M phosphoric acid, apparent pH adjusted to 4.0 with triethylamine and acetonitrile in the ration of 30:70, v/v, at a flow rate of 1.0 mL/min	225	45.05–180.2 PAR	NA	[69]
				49.2–196.98 IBU		

5	C_18_, 15 cm × 4.6 mm, 5 µm	0.01 M K_2_HPO_4_ buffer (pH 6.8) and acetonitrile in a ratio of 65:35, v/v, at a flow rate of 0.7 mL/min	222	25–100 PAR	2.30 PAR	[70]
				10–40 IBU	0.21 IBU	

6	C_18_, 15 cm × 4.6 mm, 5 µm	Acetonitrile and phosphate buffer pH 5 (20 mM) (60:40 v/v), at a flow rate of 1.0 mL/min	254	16–200 IBU	5.33 IBU	[71]
				1–30 PAR	3.30 PAR	

7	C_18_, 15 cm × 4.6 mm, 5 µm	50.0 mM phosphate buffer (pH 7.5): methanol in a gradient elution mode:	220	0.25–250	0.06 IBU	[72]
		0.01 min ⟶ 80% buffer			0.08 PAR	
		2.0 min ⟶ 80% buffer				
		5.0 min ⟶ 5% buffer				
		9.0 min ⟶ 5% buffer				
		14.0 min ⟶ 80% buffer, at a flow rate of 1.0 mL/min				

8	C_18_, 25 cm × 4.6 mm, 5 µm	Water:acetonitrile, (45:55, v/v) %, at a flow rate of 1.0 mL/min	282	10–50 both	1.60 IBU	[73]
					0.88 PAR	

9	C_18_, 15 cm × 4.6 mm, 5 µm	Acetonitrile and Na_2_HPO_4_ buffer in the ratio of 60:40 (v/v pH 7.0) at ambient temperature. Flow rate was kept at 0.8 mL/min	260	20–80 PAR	6.00 PAR	[74]
				10–70 IBU	10.00 IBU	

10	C_18_, 25 cm × 4.6 mm, 5 µm	Acetonitrile and water (30:70, v/v) + 40 mM phosphate buffer at pH 6.0 with a flow rate of 1.0 mL/min	300–330	5–25 IBU	0.013 IBU	[75]
				1–5 PAR	0.021 PAR	

11	HSS T3 10 cm × 2.1, 1.8 µm	20 mM KH_2_PO_4_ buffer (pH 7.35 with dilute phosphoric acid): acetonitrile (35:65 v/v) mobile phase at a flow rate of 0.25 mL/min	225	6.66–59.94 PAR	0.84 PAR	[76]
				8–72 IBU	0.52 IBU	

13	C_18_, 25 cm × 4.6 mm, 5 µm	Mobile phase A: purified water pH adjusted to 2.5 with orthophosphoric acid: methanol in the ratio of 950:50 (v/v) and mobile phase B: mixture of pH 2.5 buffer solution: Acetonitrile: methanol (200:200:600 v/v/v).	220	1–120 for both	NA	[77]
		0.0 min ⟶ 5% A				
		5.0 min ⟶ 5% A				
		20.0 min ⟶ 50% A				
		40.0 min ⟶ 50% A				
		55.0 min ⟶ 70% A				
		85.0 min ⟶ 70% A				
		86.0 min ⟶ 5% A				
		The flow rate of mobile phase was set as 1.0 mL/min.				

## Data Availability

The data that support the findings of this study are available in Scholar Google at https://scholar.google.com.
